# Host plants and *Wolbachia* shape the population genetics of sympatric herbivore populations

**DOI:** 10.1111/eva.13079

**Published:** 2020-09-12

**Authors:** Zhen Fu, Amanda R. Meier, Brendan Epstein, Alan O. Bergland, Carmen I. Castillo Carrillo, William R. Cooper, Regina K. Cruzado, David R. Horton, Andrew S. Jensen, Joanna L. Kelley, Arash Rashed, Stuart R. Reitz, Silvia I. Rondon, Jenita Thinakaran, Erik J. Wenninger, Carrie H. Wohleb, David W. Crowder, William E. Snyder

**Affiliations:** ^1^ Department of Entomology Washington State University Pullman WA USA; ^2^ Department of Entomology University of Georgia Athens GA USA; ^3^ Department of Plant and Microbial Biology University of Minnesota St. Paul MN USA; ^4^ Department of Biology University of Virginia Charlottesville VA USA; ^5^ Departamento de Protección Vegetal Estación Experimental Santa Catalina Instituto Nacional de Investigaciones Agropecuarias (INIAP) Quito Ecuador; ^6^ Temperate Tree Fruit and Vegetable Research USDA‐ARS Wapato WA USA; ^7^ Department of Entomology, Plant Pathology, and Nematology University of Idaho Moscow ID USA; ^8^ Northwest Potato Research Consortium Lakeview OR USA; ^9^ School of Biological Sciences Washington State University Pullman WA USA; ^10^ Malheur Experiment Station Oregon State University Ontario OR USA; ^11^ Department of Crop and Soil Science Hermiston Agricultural Research and Extension Center Hermiston OR USA; ^12^ Department of Entomology, Plant Pathology, and Nematology Kimberly Research and Extension Center University of Idaho Kimberly ID USA; ^13^ Washington State University Extension Moses Lake WA USA; ^14^ Present address: Department of Entomology Texas A&M University College Station TX USA

**Keywords:** herbivore interactions, interbreeding, pest management, plant, population genomics, *Wolbachia*

## Abstract

Changing climate and land‐use practices have the potential to bring previously isolated populations of pest insects into new sympatry. This heightens the need to better understand how differing patterns of host–plant association, and unique endosymbionts, serve to promote genetic isolation or integration. We addressed these factors in populations of potato psyllid, *Bactericera cockerelli* (Šulc), a generalist herbivore that vectors a bacterial pathogen (*Candidatus* Liberibacter solanacearum, causal pathogen of zebra chip disease) of potato (*Solanum tuberosum* L.). Genome‐wide SNP data revealed two major genetic clusters—psyllids collected from potato crops were genetically similar to psyllids found on a common weed, *Lycium* spp., but dissimilar from those found on another common non‐crop host, *Solanum dulcamara* L. Most psyllids found on *Lycium* spp. and potato represented a single mitochondrial cytochrome oxidase I (COI) haplotype that has been suggested to not be native to the region, and whose arrival may have been concurrent with zebra chip disease first emerging. The putatively introduced COI haplotype usually co‐occurred with endosymbiotic *Wolbachia*, while the putatively resident COI haplotype generally did not. Genetic intermediates between the two genetic populations of insects were rare, consistent with recent sympatry or reproductive isolation, although admixture patterns of apparent hybrids were consistent with introgression of genes from introduced into resident populations. Our results suggest that both host–plant associations and endosymbionts are shaping the population genetic structure of sympatric psyllid populations associated with different non‐crop hosts. It is of future interest to explicitly examine vectorial capacity of the two populations and their potential hybrids, as population structure and hybridization might alter regional vector capacity and disease outbreaks.

## 1 INTRODUCTION

Changing climates have the potential to shift distributions of insect species and populations, leading to abandonment of some areas and colonization of new ones (e.g., Lehmann et al., 2020; Parmesan et al., 1999; Sánchez‐Guillén, Córdoba‐Aguilar, Hansson, Ott, & Wellenreuther, 2016; Stefanescu, Penuelas, & Filella, 2009). For generalist insects which utilize multiple plant species for food sources, it may be possible to rapidly adapt to and proliferate on novel hosts as environmental change drives range shifts of the insects and/or host plants (Futuyma & Agrawal, 2009; Simon et al., 2015). Indeed, expansion of agricultural crops into new regions is believed to have underlain the emergence of several new agricultural pest insects, facilitating shifts from native to agricultural host plant species (Crossley, Rondon, & Schoville, 2019; Jiggins & Bridle, 2004). Host shifts can sometimes result in sympatric, but genetically isolated, herbivore populations that can eventually form distinct species (Jiggins & Bridle, 2004). So, both natural and human‐facilitated movement of plants and herbivorous insects can have important implications for pest management and the emergence of new pests. Recent advances in population genomics now make it possible to track genetic isolation and hybridization of sympatric insect species and populations at a relatively fine scale; this in turn can help pest management by allowing the inference of pest movement patterns across landscapes and among crop and non‐crop host plants (e.g., Angelella, Michel, & Kaplan, 2019; Barman, Parajulee, Sansone, Suh, & Medina, 2012; Fu et al., 2017).

Interactions between sympatric herbivore populations may also be mediated by endosymbionts such as *Wolbachia*. *Wolbachia* spreads by providing infected females with a reproductive advantage (Werren, Baldo, & Clark, 2008). The mitochondrial genomes of infected individuals can also “hitchhike” with *Wolbachia* and replace the mitochondrial COI haplotypes in infected individuals (Narita, Nomura, Kato, & Fukatsu, 2006; Schuler et al., 2016; Werren et al., 2008). The ability of *Wolbachia* to affect hosts and sweep through insect populations has led to interest in using *Wolbachia* to indirectly suppress the incidence of vector‐borne pathogens (e.g., Hoffmann, Ross, & Rašić, 2015; McGraw, Merritt, Droller, & O’Neill, 2002; McMeniman et al., 2009; Schmidt et al., 2017). However, the success of such applications relies on understanding the dynamics of naturally occurring sweeps of *Wolbachia*, and few studies have evaluated effects of *Wolbachia* on the population genetics of insect vectors in the field (Chu, Gill, Hoffmann, & Pelz‐Stelinski, 2016; Krstić et al., 2018).

The potato psyllid, *Bactericera cockerelli* (Šulc) (Hemiptera: Triozidae), is a small, multivoltine, phloem‐feeding insect that uses host plants across multiple genera within the Solanaceae. In its western North American range, *B. cockerelli* comprises several unique COI haplotypes that differ in host use and endosymbionts (Cooper et al., 2015; Fu et al., 2017; Swisher et al., 2013b). Potato psyllids transmit a bacterial pathogen (“*Candidatus* Liberibacter solanacearum”, LSO, syn. “*Ca*. L. psyllaurous”), which causes zebra chip disease in potato plants (*Solanum tuberosum* L.) (Hansen, Trumble, Stouthamer, & Paine, 2008). The zebra chip pathogen was first detected in the major potato growing regions of the U.S. states of Washington, Oregon, and Idaho in the summer of 2011, following its initial discovery in Texas and northern Mexico 20–25 years earlier (Munyaneza, 2015). Two common psyllid COI haplotypes occur in these northwestern growing regions, a “western” type found in these three states in 2008 and suspected of being a recent arrival (Munyaneza, Crosslin, & Buchman, 2009; Swisher, Munyaneza, & Crosslin, 2013a), and an apparently resident “northwestern” type that has yet to be collected outside of this region (Swisher et al., 2013a). Psyllids of the western COI haplotype are more likely to harbor the pathogen than the northwestern COI haplotype, suggesting LSO emergence in the northwestern US may have been associated with the invasion of western psyllids (Swisher et al., 2013a, 2014). Moreover, only western psyllids, but not northwestern psyllids, appear to harbor *Wolbachia* (Cooper et al., 2015), which may increase fitness and promote gene introgression into resident populations. Indeed, under laboratory conditions, eggs resulting from matings between *Wolbachia*‐infected western COI‐haplotype females and uninfected northwestern COI‐haplotype males are viable, whereas only 2% of eggs from reciprocal matings are viable (Cooper et al., 2015). Thus, the presence of *Wolbachia* in western psyllids would drive the dynamics of the hybridization with sympatric northwestern psyllids.

**Figure 1:**
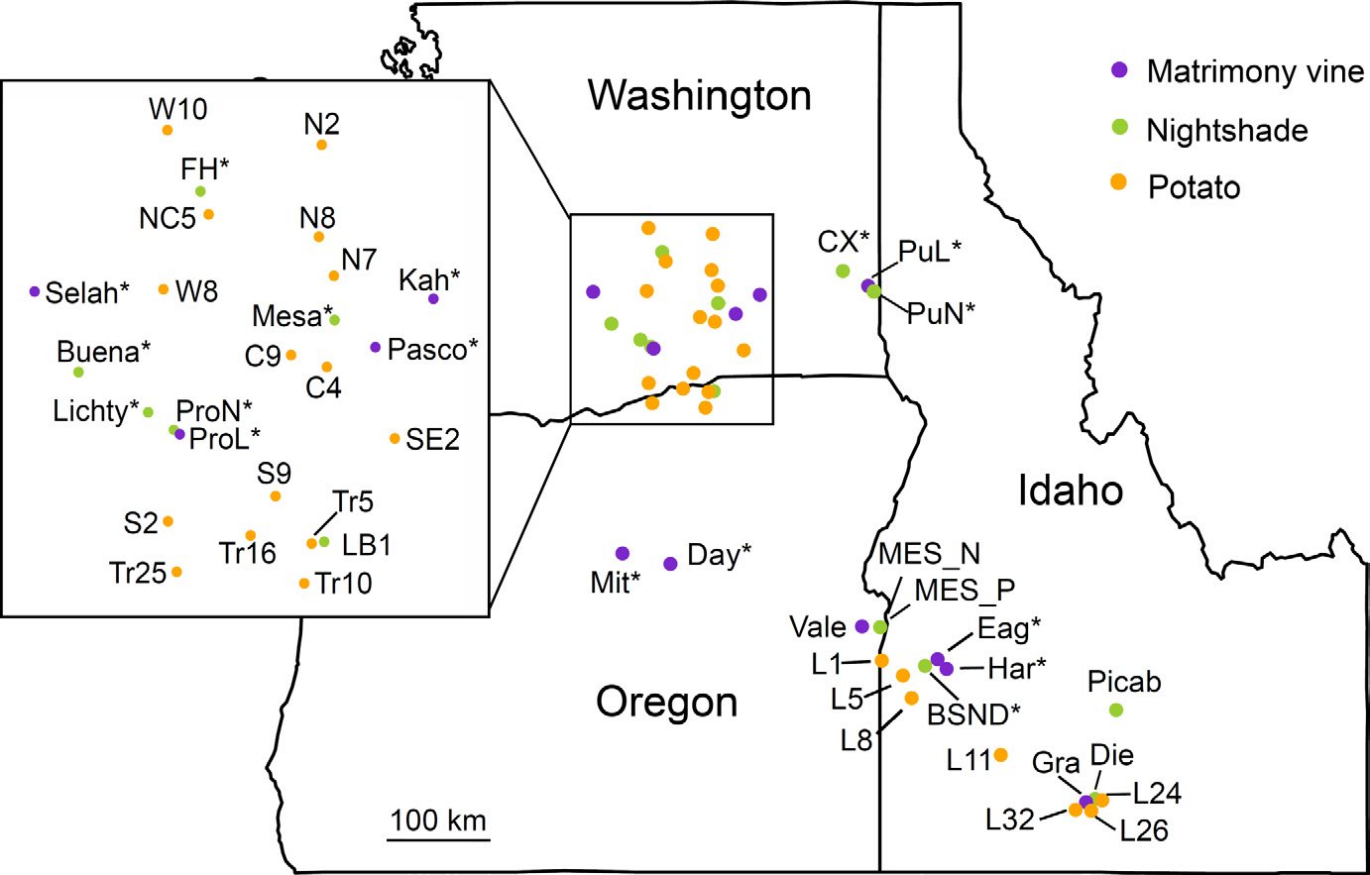
Potato psyllid sampling sites across the US states of Washington, Oregon, and Idaho. Potato psyllids were collected from matrimony vine (purple), bittersweet nightshade (green), or potato (orange). *indicates non‐crop host (matrimony vine and bittersweet nightshade) sites that were sampled pre‐ and post‐season

Host plant preference might differ between the haplotypes (Cooper, Horton, Miliczky, Wohleb, & Waters, 2019; Fu et al., 2017; Swisher et al., 2013b), which could reinforce genetic isolation. In this region, psyllids occur not only on potato, where they are of agricultural interest, but also on the non‐native perennial plants bittersweet nightshade (*Solanum dulcamara* L.) and matrimony vine (*Lycium* spp.). While potato production is focused within two areas, one in southcentral Washington/northcentral Oregon and another in southern Idaho, these perennial hosts are distributed broadly over the entire region (Figure 1). These weeds are thought to serve as “bridge” hosts for psyllids; psyllids may move from bittersweet nightshade or matrimony vine plants to nearby potato fields in the spring and summer, and then return once the potato plants have been harvested (Horton et al., 2015b, 2016). However, evidence for these patterns of seasonal movement is largely circumstantial, such that it is unclear whether one or both host plants are the source of psyllids found in potato fields each summer (Horton et al., 2016). Furthermore, it is unclear whether the western and northwestern psyllid haplotypes indeed differ in their host plant use in the region. Because COI is maternally inherited, the degree of hybridization between the two psyllid types has not been determined, and these two competing scenarios—introgression or reproductive isolation—have not been assessed.

Here, we explore how host use patterns and endosymbiont communities affect the population genetic structure of psyllid populations across three host plants. First, we assessed whether the genetic structure of psyllid populations is consistent with both the recent arrival of genetically distinct psyllids from outside of the growing region and the seasonal movement of psyllids among host plants. Second, we address whether the resident (northwestern) and putatively introduced (western) psyllids differ in their host–plant associations or carry distinct endosymbionts. Third, we evaluate whether hybridization between genetically distinct psyllids occurs frequently. Our ultimate goal is to inform how the arrival of new psyllid genetic types might have influenced the sudden emergence of zebra chip disease in potato. Because our work was conducted at a broad geographic scale (Figure 1), we hope to provide a model for assessing how host use, population genetics, and endosymbionts might interact in vector‐borne pathosystems more generally.

## 2 MATERIALS AND METHODS

### 2.1 Region‐wide psyllid collection and DNA extraction

Psyllids were collected from 23 potato, 12 bittersweet nightshade (*Solanum dulcamara* L), and 11 matrimony vine (*Lycium* spp.) sites across three northwestern US states in 2016 (Figure 1, Table S1). Collection sites for the non‐crop hosts were largely opportunistically identified and most often found near major highways, given the rugged mountainous terrain of the region, association of the weedy host plants with human‐mediated disturbance, and challenges associated with gaining permission for collections on remote private land. We sampled non‐crop hosts at the beginning (“pre‐season,” May–June) and end of the potato growing season (“post‐season,” September–October); potato crops were sampled once in mid‐season (July–August). This captured key periods when psyllids might move from non‐crop hosts to crops and back again (Horton, Cooper, Munyaneza, & Swisher, 2015a). Psyllids were collected using a suction sampler or beat sheets and placed on dry ice for transport; at the laboratory, psyllids were placed in 95% ethanol in a −20°C freezer.

From each collection site and date, four to ten adult psyllids were haphazardly selected for DNA extraction (total *N* = 566). We used the DNeasy Blood & Tissue kit to extract psyllid DNA (Qiagen, Germantown, MD) by placing single insects in a microcentrifuge tube with 180 µl ATL buffer. We ground the insect tissue submerged in buffer for 1 min using a pestle driven by a MicroTube Homogenizer (VWR, Radnor, PA); otherwise, the extraction was conducted following the kit protocol. One hundred µl AE buffer from the kit was used to elute DNA. The quantity of DNA was measured using a Qubit 3.0 Fluorometer (Thermo Fisher Scientific).

### 2.2 NextRAD sequencing and COI haplotyping

DNA samples were sent to SNPsaurus LLC (Eugene, OR) for NextRAD library preparation and sequencing as described in Fu et al. (2017). DNA was fragmented with the Nextera reagent (Illumina), which ligated short adapters to the fragmented DNA. DNA fragments were then amplified with two primers that matched adapter sequences, with one extended an additional nine nucleotides (GTGTAGAGC), so only fragments that hybridized to the selective sequence were amplified. The NextRAD libraries were sequenced on an Illumina HiSeq4000 with 1 × 150 base pairs (bp) configuration to generate 94 × coverage per individual at each locus.

We characterized the COI haplotype of each sample following the high‐resolution melting curve method (Swisher, Munyaneza, & Crosslin, 2012). High‐resolution melting is a post‐PCR analysis that detects small variation in DNA sequences, utilizing double‐stranded DNA binding dye and a quantitative PCR instrument to capture the signature curve of samples while PCR products are being heated at precise increments. For psyllids whose melting curve signal differed from standards, we used Sanger sequencing to confirm the COI sequence. We used a Pearson's chi‐squared test in R (R Core Team, 2016) to evaluate whether the frequency of haplotypes varied among host plant species and across the geographic spread of our sampling network.

### 2.3 Sequence alignment, variant calling, and filtering

Quality trimming of raw reads and variant calling was conducted by SNPsaurus. Genotyping analysis used custom scripts (SNPsaurus) that trimmed reads using bbduk (Bushnell, 2014) with parameters: ktrim = r; k = 17; hdist = 1; mink = 8; minlen = 100; ow = t; qtrim = r; and trimq = 10. Next, a *de novo* reference was created by collecting 10 million total reads, evenly from all of the samples, and excluding contigs that had fewer than 7 or more than 700 mapped reads. The remaining loci were then aligned to each other to identify allelic loci and collapse allelic haplotypes to a single representative haplotype. All reads were mapped to the reference with an alignment identity threshold of 95% using bbmap (Bushnell, 2014). Genotype calling was done using “mpileup” in SAMtools and bcftools (Li et al., 2009). The vcf file was filtered to remove alleles with a minor allele frequency of <3% across the dataset. Loci that were heterozygous in all samples or had more than two alleles in a sample (suggesting collapsed paralogs) were removed.

We removed 37 samples with mean coverage < 5× and 24 samples with > 30% missing loci. Thus, the final dataset contained 505 samples. We further filtered the 9,180 SNPs from SNPsaurus. First, we used VCFtools (Danecek et al., 2011) to calculate coverage, heterozygosity, allele frequencies, and the proportion of missing data. Then we removed 2,082 loci that were missing in >15% of individuals, and 111 loci with observed heterozygosity >0.5. We kept only single nucleotide polymorphisms by removing all indels. We used VCFtools to test Hardy–Weinberg equilibrium (HWE) for each locus in every population with sample size ≥8. HWE tests were applied only to the loci with no missing genotypes, with a set *P*‐value cutoff of 0.001; 46 loci that violated HWE in ≥20% of populations were removed. To ensure that loci used in downstream analyses were approximately independent, we randomly sampled one variant from each of the 1,835 contigs. Finally, we identified loci potentially under selection using LOSITAN (Antao, Lopes, Lopes, Beja‐Pereira, & Luikart, 2008) and BayeScan (Foll & Gaggiotti, 2008), with a *q*‐value of 0.1. We removed 44 loci putatively under selection detected by both programs. Thus, altogether, the final dataset contained 1,791 SNPs.

### 2.4 Population genomics analyses

#### 2.4.1 Population structure

We used four analyses to characterize psyllid population structure. First, we used SplitsTree (v4; Huson & Bryant, 2006) with default settings to construct a neighbor‐net from a genetic distance matrix based on pairwise differences per base pair. The neighbor‐net algorithm produces a graph with reticulations representing recombination or uncertainty. Second, we used ADMIXTURE (v1.3; Alexander, Novembre, & Lange, 2009) to identify genetically distinct psyllid groups by assessing clustering in the data. We did this because COI haplotypes only reflect a small proportion of maternal genetic information, while ADMIXTURE uses genome‐wide markers. We performed 50 runs from different random seeds for each predefined *K* (1 < *K* ≤20), which represents the number of putative ancestral populations. ADMIXTURE estimates the proportion of putative ancestry for each individual based on its genotype. We conducted ADMIXTURE runs in “unsupervised” mode because psyllid gene flow could occur among host plants and study sites. Ancestry coefficients from 50 runs were aligned and averaged using CLUMPAK (Kopelman, Mayzel, Jakobsson, Rosenberg, & Mayrose, 2015). Third, we conducted a principal component analysis (PCA) of the genome‐wide SNPs using the smartpca algorithm in EIGENSOFT (Price et al., 2006) to visualize population structure. Fourth, we used RADpainter and fineRADstructure (Malinsky, Trucchi, Lawson, & Falush, 2018) to infer the fine population structure of psyllids characterized as northwestern or western COI haplotypes. For ease of visualization and interpretation, we limited this evaluation to 25% (124/495) of the total pool of psyllids that were characterized as western and northwestern haplotype. We intentionally selected these 124 psyllids because they were spatially dispersed in the initial principal components analysis (PCA) along PCA1 (described below) as we attempted to avoid sampling a subset of psyllids that had low genetic diversity.

**Figure 2:**
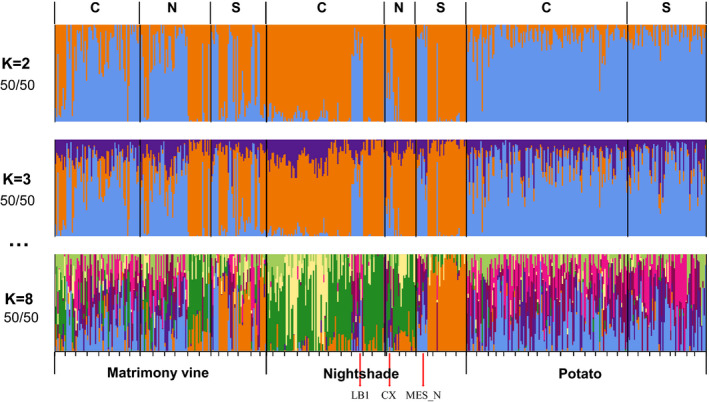
ADMIXTURE of potato psyllids separated by hosts. Orange bars represent the NW genetic group and blue bars represent the W genetic group. K (the number of putative ancestral populations) = 8 was the optimal K as it minimized the cross‐validation error. Numbers below each K indicate the number of runs (out of 50) that showed the representative grouping. Sampling region is labeled along the top: C = Columbia Basin of Oregon and Washington, N = non‐potato growing region, and S = Snake River Plain of Idaho. The black vertical bars in each panel divide psyllids collected from different growing regions. The tick marks in K = 8 separate each population (combination of sampling date and locality). Samples from LB1, MES_N, and CX were genetically similar to potato psyllids from potato fields

To further identify potentially admixed individuals, we ran an *f*_3_ statistic using the program ADMIXTOOLS (Patterson et al., 2012). The *f*_3_ statistic can be used to test whether a target population (C) is admixed between two source populations A and B (C; A, B). In our instance, we were interested in examining individual psyllids that were most likely to be hybrids that were derived from the putatively introduced and resident populations. We picked two source populations based on the membership coefficients generated in ADMIXTURE (*K* = 2). We assumed that psyllids with 100% assignment to the lineage corresponding to the W haplotype (blue color in Figure 2, totaling 47 individuals) would be the closest proxies to represent the putatively introduced psyllid lineage; conversely, psyllids assigned 100% to the other lineage (orange color in Figure 2, totaling 80 individuals) could be used as proxies for a putatively resident psyllid population.

#### 2.4.2 F‐statistics and spatiotemporal separation

As noted above, psyllids might move each year from either bittersweet nightshade or matrimony vine, or both non‐crop hosts, to potato fields each summer before then returning to overwinter (Castillo Carrillo, Fu, Jensen, & Snyder, 2016; Horton et al., 2015a; Wenninger, Dahan, Thornton, & Karasev, 2019). These movement patterns, if they occur, could lead to increasing relatedness through the season for psyllids on some subset of the host plants. We assessed this by determining pairwise population differentiation (*F*_ST_) between any pair of populations from a non‐crop site (with pre‐ and post‐season samples) and a potato site in the same region. This allowed for a comparison of genetic differentiation between pre‐ and post‐season populations in relation to potato. *F*_ST_ was estimated using the package StAMPP in R (Pembleton, Cogan, & Forster, 2013). We calculated *F*_ST_ for populations with sample size ≥8.

#### 2.4.3 Analysis of Molecular Variance (AMOVA)

We performed AMOVA to determine how much genetic variation is explained by sampling sites and host plant species relative to the major ADMIXTURE genetic groups (*K* = 2) determined by majority rule. Sampling sites were nested within host plant species and nested within genetic groups (see *Population genomic patterns*). The function poppr.amova in R package poppr was used to conduct AMOVA (Kamvar, Brooks, & Grünwald, 2015; Kamvar, Tabima, & Grünwald, 2014).

#### 2.4.4 Multiple matrix regression with randomization (MMRR)

To further examine correlations between genetic distance (*F*_ST_) and other independent variables, for example, host plant, COI haplotypes, and geographic distance, we conducted MMRR (Wang, 2013). We created four matrices, including geographic distance, *F*_ST_, host plant, and COI haplotype, and we ran MMRR using the R package tseries (Wang, 2013). For details of matrix building, please refer to supporting information.

### 2.5 16S ribosomal RNA (rRNA) gene sequencing

To characterize the bacterial microbiota of individual psyllids, 77 insects that had sufficient DNA remaining after NextRAD sequencing were processed for 16S rRNA gene sequencing. Primers flanking the V3‐V4 hypervariable regions of the 16S rRNA gene (341F: 5′‐AGC CTA CGG GNG GCW GCA G‐3′; 806RB: 5′‐CCG GAC TAC NVG GGT WTC TAA T‐3′) (Chakravorty, Helb, Burday, Connell, & Alland, 2007) were used in a two‐step, dual‐barcoded PCR procedure. The first PCR was composed of 0.25 µl of each 10 mM forward and reverse primers, 5 µl 10 × PCR buffer, 6 µl 25 mM MgCl_2_, 1 µl 10 mM dNTPs, 0.6 µl BSA (20 mg/ml), 0.25 µl Taq DNA polymerase (Thermo Fisher Scientific, Waltham, MA), 20 µl of psyllid DNA, and 16.65 µl molecular grade water to reach 50 µl. The PCR was denaturation at 95°C for 3 min, followed by 25 cycles of 95°C for 30 s, 51°C for 30 s and 72°C for 1 min; a final extension was at 72°C for 10 min. PCR products were visualized on 1.5% agarose gels. Samples with a clear band of ~460 bp were processed in the second PCR, which included 4 µl 10 × PCR buffer, 7.2 µl 25 mM MgCl_2_, 1.2 µl BSA, 0.8 µl 10 mM dNTPs, 0.4 µl Taq DNA polymerase, 3 µl barcode primers, 10 µl of five time diluted first PCR products, and 13.4 µl molecular grade water. Dual barcode primers were ordered from IBEST (University of Idaho, USA), and each psyllid sample processed here received a unique barcode. The second PCR was denaturing at 95°C for 3 min, followed by 10 cycles of 95°C for 30 s, 60°C for 30 s, and 72°C for 1 min; the extension was at 72°C for 10 min. Samples showed a single band at the expected size of ~580 bp during gel electrophoresis. Equal molar of PCR amplicons from the second PCR were pooled and sequenced on Illumina MiSeq with a 2 × 300 bp configuration.

### 2.6 Microbiome data analysis

Raw sequences were de‐multiplexed based on barcodes. Paired‐end reads were trimmed using Trimmomatic (Bolger, Lohse, & Usadel, 2014) with the parameters “LEADING:20 TRAILING:20 SLIDINGWINDOW:5:15 MINLEN:200,” and paired reads were merged using Flash (Magoč & Salzberg, 2011). We cut merged reads to 403 bp to fit the shortest sequence using Trimmomatic. Next, we used the R package DADA2 (Callahan et al., 2016) to conduct analyses including error rate learning, de‐replication, sequence table construction, and taxonomy assignment of unique sequences. The algorithm of DADA2 differs from other programs which consolidate sequences that are dissimilar within a fixed threshold (often 3%) and construct unique operational taxonomic units (OTUs). Instead, DADA2 infers sequences exactly and resolves sequence differences as low as a single nucleotide, which is a more suitable algorithm for this study to assess fine‐scale variation of endosymbiont strains. We filtered out contaminating sequences (e.g., chloroplasts and mitochondria) and removed bacterial taxa that were not characterized to Class. To focus on relatively abundant taxa, we removed taxa with relative abundance <1%, taxa that received <5 reads, or taxa that showed up in <3 samples. To further verify the characterization of the bacterial taxa, we manually queried the unique bacterial sequences using BLASTN (Altschul, Gish, Miller, Myers, & Lipman, 1990) against the nr/nt database. Downstream analysis was conducted using the R package phyloseq (McMurdie & Holmes, 2013), including taxa and sample filtering, sequencing depth scaling, and quantifying taxa abundance. We evaluated differences in endosymbiont communities among psyllid ADMIXTURE genetic groups and host plant species using permutational multivariate ANOVA (PERMANOVA). We used the adonis function in the vegan package and calculated dissimilarities among samples using the Bray–Curtis metric for PERMANOVA (Oksanen, et al., 2019). In addition, we checked for homogeneity of dispersion among groups using the betadisper function in vegan.

## 3 RESULTS

### 3.1 COI haplotyping

Among the 566 psyllids we examined, 359 (63.4%) and 191 (33.7%) were of the dominant western and northwestern COI haplotypes, respectively, and 16 (2.8%) were other COI haplotypes (2 of the southwestern COI haplotype [0.4%], 7 of the central COI haplotype [1.2%], and 7 that were inconclusive [1.2%]). Different COI haplotypes generally were associated with different host plant species (only considering northwestern and western COI haplotypes, Pearson's chi‐squared test: *χ* ^2^ = 259.73, *df* = 2, *p* < 2.2e^−16^), where 76.2% of psyllids on bittersweet nightshade were of the resident northwestern COI haplotype, and 99.5% of psyllids on potato were of the putatively introduced western COI‐haplotype type. Of the psyllids collected from matrimony vine, 65% were western COI haplotype and 31.7% were northwestern COI haplotype. The frequency of COI haplotypes did not vary between the two potato growing areas in our study region (southcentral Washington/northcentral Oregon versus southern Idaho in Figure 1; Pearson's chi‐squared test, *χ* ^2^ = 0.1857, *df* = 1, *p* = .67).

### 3.2 Population genomic patterns

**Figure 3:**
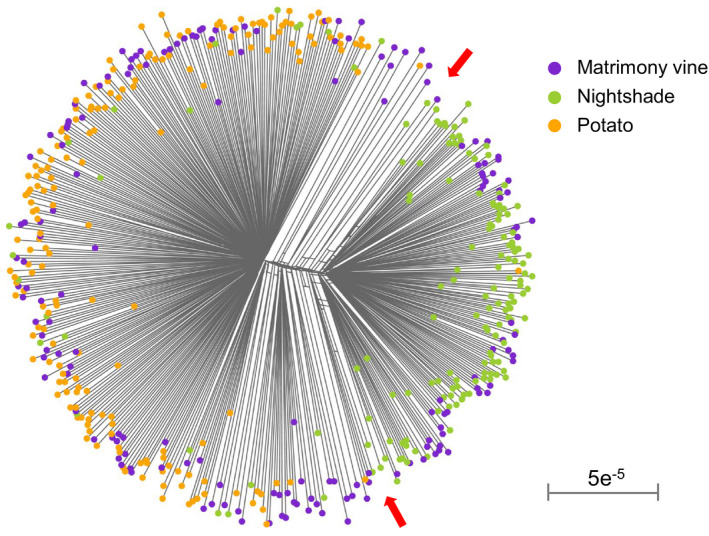
Neighbor‐net clustering of potato psyllids sampled from three plant hosts: matrimony vine, bittersweet nightshade, and potato. Scale unit is number of differences per base pair. Arrows delimit bittersweet nightshade and matrimony vine/potato clusters

Neighbor‐net analysis indicated that 70.1% of matrimony vine‐collected psyllids clustered with potato‐collected psyllids, while only 14.8% of bittersweet nightshade‐collected psyllids clustered with potato‐collected psyllids (Figure 3). Moreover, 30% of matrimony vine‐collected psyllids clustered with bittersweet nightshade‐collected psyllids, demonstrating that psyllids from matrimony vines were mixed in genetic composition (Figure 3).

We found a similar pattern in ADMIXTURE as with the neighbor‐net clustering analysis. The cross‐validation error was minimized at eight clusters (*K* = 8). However, individuals were highly admixed, and the results were not easily interpretable. Thus, we focus on the results at lower *K* values. Most bittersweet nightshade‐ and potato‐collected psyllids were assigned to different groups at *K* = 2, while psyllids collected from matrimony vine were assigned to both groups (Figure 2). ADMIXTURE estimates membership coefficients for each sample, which sum to 100%. Thus, we used the assignment at *K* = 2 in ADMIXTURE to describe the two major genetic groups based on majority rule. Genetic group 1 (orange bars in Figure 2) included 85.2% of psyllids from bittersweet nightshade and 37.8% of psyllids from matrimony vine. Genetic group 2 (blue bars in Figure 2) included 98.4% of potato‐collected and 62.2% of matrimony vine‐collected psyllids. The two genetic groups corresponded roughly to the two predominant COI haplotypes in the region, the northwestern (orange, group 1) and western (blue, group 2), with the exception of 21 samples that may be evidence of admixture between the two otherwise‐distinct genetic groups (see *Evidence of admixture between two lineages*, below). To avoid confusion, we will henceforth refer to genetic groups assigned by majority rule in ADMIXTURE while *K* = 2 as W and NW ADMIXTURE genetic groups.

Although the majority of psyllids from bittersweet nightshade were placed in a different genetic group than those from potato, dozens of psyllids from three bittersweet nightshade sites were genetically similar to psyllids from potatoes. Among them, sites LB1 and MES_N were in close proximity to potato fields (Figure 1). Conversely, site CX was not in a potato growing region (>50 km away from closest potato field). *K* = 3 identified modest genetic divisions among growing regions (Figure 2) compared with genetic variation among host plants and genetic groups.

### 3.3 Evidence of admixture between two lineages

**Figure 4:**
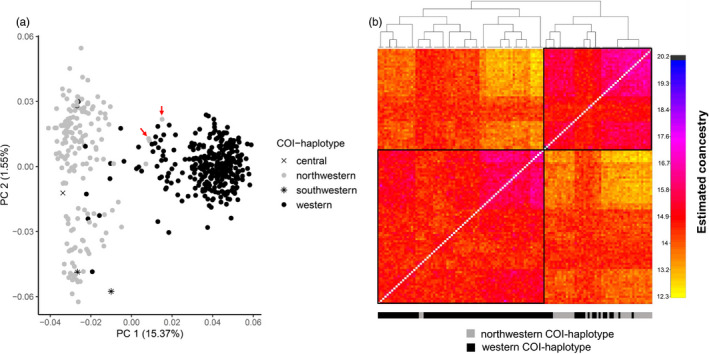
(a) Principal component (PC) 1 and PC2 of the genome‐wide SNPs of potato psyllids colored by COI haplotype. Red arrows indicate two northwestern COI haplotypes that clustered with western COI‐haplotype psyllids. (b) fineRADstructure clustering and coancestry matrix for 124 potato psyllids that were characterized as NW or W COI haplotypes. Coancestry measures genetic similarity of two individuals; higher coancestry (colder color) indicates less divergence of two individuals

There was evidence of admixture of the resident and putatively introduced psyllid populations, primarily in the direction of western COI‐haplotype females mating with northwestern COI‐haplotype males. A PCA generated from all 505 psyllids using genome‐wide SNPs indicated that 27 psyllids characterized as the western COI haplotype grouped with northwestern COI‐haplotype psyllids (Figure 4a). The fineRADstructure analysis performed on a subset of the samples (124 psyllids) also revealed that 19 psyllids with the western COI haplotype clustered in the clade mainly composed of northwestern COI‐haplotype psyllids. In contrast, only two northwestern COI‐haplotype psyllids clustered in the clade composed of mostly insects with the western COI haplotype (Figure 4b; Table S2). As depicted on the PCA plot, these two northwestern COI haplotypes also clustered with western haplotype psyllids (Figure 4a). Similarly, examination of ADMIXTURE assignment (*K* = 2) showed that 20 insects (e.g., W105, Picabo3, and Har_Jun_2) assigned to the NW ADMIXTURE genetic group were of the western COI haplotype (Table S2), but only two insects assigned to the W ADMIXTURE genetic group were of the northwestern COI haplotype. Using the *f*_3_ statistic, 17 psyllids were detected as hybrids (Figure S1). Among these 17 psyllids, six individuals were also flagged as possible hybrids from at least one of the three tests described above (fineRADstructure, ADMIXTURE, and PCA).

### 3.4 AMOVA and MMRR

ADMIXTURE genetic grouping explained >20% of genetic variation (Table 1), but the largest component of genetic variability was explained at the individual level and within sites (Table 1). Within each genetic group, host plant accounted for <1% of genetic variation.

**Table 1:** Results of analyses of molecular variance (AMOVA) of potato psyllid samples grouped by different factors

Variance %	Φ‐statistics	*p*‐value	Component
21.59	0.216	^*^	Between ADMIXTURE genetic groups^a^, ^*^
0.08	0.001	.272	Between hosts within ADMIXTURE genetic groups
2.28	0.029	.001	Between sites within hosts
23.37	0.307	.001	Between samples within sites
52.68	0.473	.001	Within individuals

^a^ ADMIXTURE genetic group was defined based on majority rule with *K* = 2.

* *p*‐value for this component was not assessed. As the ADMIXTURE clusters were identified based on the same data the AMOVA was performed on, the *p*‐values would not be meaningful (Meirmans, 2015)

With 41 populations included in MMRR, we conducted analyses with all combinations of the explanatory variables (a) geographic distance, (b) host plant identity, and (c) COI haplotype; genetic distance (*F*_ST_) was the response. Geographic distance alone explained very little genetic distance (*r* ^2^ ~ 0.02). COI haplotype and host plant explained more (*r* ^2^ = 0.652 and 0.226, respectively) (Table S3).

### 3.5 Population differentiation and temporal genetic variation

**Figure 5:**
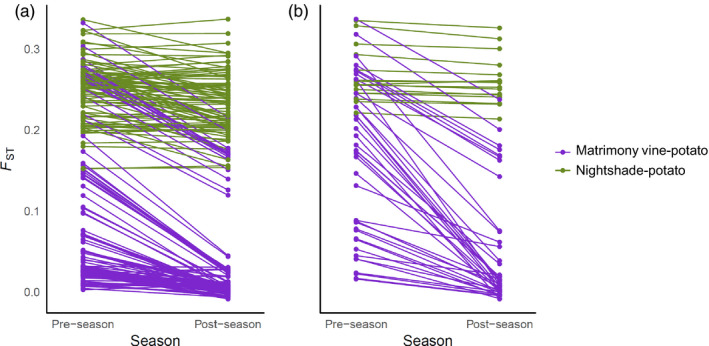
Pairwise *F*_ST_ between potato psyllids from non‐crop host sites and potato sites within (A) Columbia Basin and (B) Snake River Plain. For each non‐crop site where two insect collections were conducted, we estimated *F*_ST_ between the non‐crop site and a potato field within the same region, both pre‐ and post‐season. The line connects the pre‐season *F*_ST_ and post‐season *F*_ST_ for the non‐crop host site

Psyllid populations turned over at many matrimony vine sites from pre‐ to post‐season, indicated by *F*_ST_ changes of the pre‐ and post‐season populations (Table S4, Figure 5). Post‐season psyllid populations from matrimony vine were more genetically similar to psyllids from potatoes than pre‐season psyllid populations (Figure 5). In contrast, psyllids from bittersweet nightshade sites did not become more genetically similar to psyllids from potato sites from pre‐ to post‐season (Figure 5, Welch Two sample *t*‐test of pre‐ and post‐season *F*_ST_ differences of two host‐potato comparisons, *t* = 3.4362, *df* = 166.98, *p* < .001). Population turnover at matrimony vine sites was also reflected in genetic group composition change. Sites with high *F*_ST_ between pre‐season and post‐season populations often exhibited a shift from the NW ADMIXTURE genetic group‐dominant to W ADMIXTURE genetic group‐dominant (e.g., sites Eag, Har, and Selah; Table S4).

### 3.6 Psyllid endosymbiont communities

**Figure 6:**
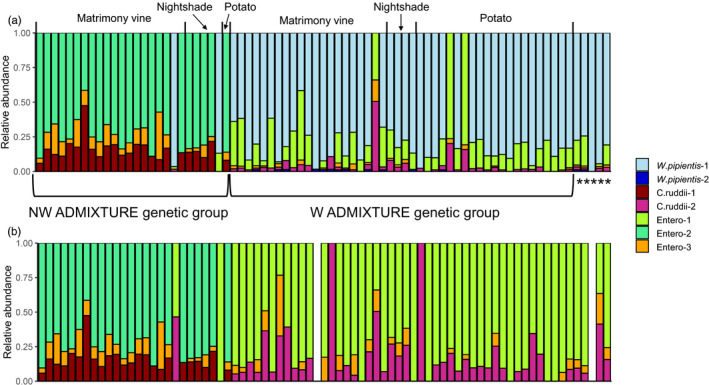
(a) Microbiome of potato psyllids separated by genetic groups, including all seven bacterial strains detected. Genetic group corresponds to the group assignment in ADMIXTURE (*K* = 2). Each color represents a bacterial strain. Due to limitations of 16S rRNA gene sequencing, we could not characterize Enterobacteriaceae strains to species level. (b) Microbiome of potato psyllids separated by genetic groups, with *Wolbachia pipientis* excluded. * Indicates samples without genotyping information due to low‐quality reads

Over 6 million paired‐end reads (300 bp length, 5,275–108,865 reads per sample) were generated from the 16S rRNA sequencing of 77 samples. We focused on the taxa that were most prevalent in the dataset instead of describing taxa that occurred in a few samples with low reads. After filtering (see filtering criteria in “Material and Methods”), seven bacterial strains were retained in the final dataset (Figure 6). Two strains were *Wolbachia pipientis*: the most dominant *W. pipientis* strain (present in 50/77 samples with high abundance, Table S6), *W. pipientis‐1*, was 100% identical to a known *W. pipientis* 16S ribosomal RNA gene (GenBank accession: AF501664.1). The less abundant *W. pipientis* (present in 14/77 samples with low abundance, Table S6), *W. pipentis*‐2, was only one nucleotide different from *W. pipentis*‐1 (Table S4). Two strains were *Candidatus* Carsonella ruddii. The strain associated with the NW ADMIXTURE genetic group (present in 24/77 samples), *C. ruddii*‐1, was 100% identical to a known *C. ruddii* gene (KR045612.1). The strain associated with the W ADMIXTURE group, *C. ruddii*‐2, differed from *C. ruddii*‐1 by three nucleotides. The remaining three strains were unclassified and could only be identified to the Enterobacteriaceae family (Figure 6, Table S5). *Candidatus* Liberibacter was not detected in the microbiome data. Overall, the composition of psyllid endosymbiont communities varied strongly between genetic groups (PERMANOVA, *df* = 1, *F*‐statistic = 193.77, *p* = .001). We were unable to evaluate variation in psyllid endosymbiont communities among host plant species because of uneven sample sizes leading to heterogeneity of variance (9 psyllids from bittersweet nightshade, 45 psyllids from matrimony vine, and 25 psyllids from potato, beta‐dispersion, *df* = 2, *F* = 11.203, *p* = .001).

As expected, 93% of psyllids in the W ADMIXTURE genetic group contained *W. pipientis*, whereas psyllids in the NW ADMIXTURE genetic group rarely (7.4%) harbored *Wolbachia*. The two *Wolbachia*‐infected psyllids in the NW ADMIXTURE genetic group were of the western COI haplotype, suggesting they were the product of successful matings between infected western COI‐haplotype females and uninfected northwestern COI‐haplotype males. Interestingly, differences in endosymbiont communities between genetic groups were not driven solely by *Wolbachia*; endosymbiont community structure remained distinct between the ADMIXTURE genetic groups when *Wolbachia* was removed from analyses (Figure 6b). All psyllids carried *Carsonella*, although a few taxa were only characterized to the genus level with very low abundance (<1%). Two highly abundant *C. ruddii* strains were present in all psyllids except two, with ADMIXTURE genetic groups containing distinct strains (Figure 6). In addition, psyllids in each ADMIXTURE genetic group carried two Enterobacteriaceae strains; one strain (Entero‐3) was shared between ADMIXTURE genetic groups and another strain was unique to each group (Figure 6).

## 4 DISCUSSION

Our population genomic approach, along with mitochondrial COI haplotyping, allowed us to examine the genetic structure and infer movement patterns of a plant pathogen vector, the potato psyllid. Consistent with either recent sympatry of the western and northwestern genetic types in the northwestern US, or longer‐term sympatry with reproductive isolation, there was limited evidence of gene flow between psyllid genetic types (<3% of individuals out of 505 psyllids analyzed). Psyllid genetic types also varied in their host use; potato fields harbored primarily W ADMIXTURE genetic type psyllids, and these psyllids used mainly matrimony vine, not bittersweet nightshade, as non‐crop hosts. While the results of hybridization between genetically distinct psyllids was not observed frequently, the genetic makeup of hybrids is consistent with the presence of *Wolbachia*‐induced cytoplasmic incompatibility resulting in *Wolbachia* and associated haplotypes being present in the resident psyllid genetic group. Our results suggest that both host–plant associations and endosymbionts are shaping genetic segregation/integration of the two psyllid sub‐populations.

We found that the W and NW ADMIXTURE genetic groups are distinct populations with limited gene flow, which could be consistent with the hypothesis that western psyllids have dispersed into the region recently (Horton et al., 2015b; Nelson, Swisher, Crosslin, & Munyaneza, 2014). Furthermore, potato crops were colonized only by W ADMIXTURE genetic group psyllids, consistent with the first zebra chip outbreaks in the region being spurred by the movement of infected western psyllids into potato fields, not by resident northwestern psyllids. Psyllid genetic groups differed strongly in their use of non‐crop plants, with the W ADMIXTURE genetic group primarily using matrimony vine and the NW ADMIXTURE genetic group primarily using bittersweet nightshade. This separation in host use has been shown elsewhere to maintain genetically distinct populations (Drès & Mallet, 2002; Ferrari, West, Via, & Godfray, 2012), although the two psyllid genetic groups do co‐occur on both non‐crop host species, providing opportunities for hybridization. To better understand the extent of gene flow among psyllid genetic groups within the species more broadly, future work should sample regions beyond the northwestern US; this in turn might yield insight into the initial spread of the zebra chip pathogen northward from its apparent origin point in Mexico (Horton et al., 2015b).

The association of the W ADMIXTURE genetic group with matrimony vine and potato may reflect the phenology of these two hosts. Matrimony vine plants have a leaf flush in the spring, followed by a leaf drop in the summer, occurring at the time when psyllids first begin arriving in irrigated potato crops (Horton et al., 2015a; Thinakaran et al., 2017). Matrimony vine plants then have a second leaf flush in the fall that coincides with potato harvest, providing a host for displaced psyllids (Thinakaran et al., 2017). In contrast, bittersweet nightshade plants maintain their foliage through the summer (Castillo Carrillo et al., 2016), and psyllids on these plants appear to rarely migrate to or from potato. In addition, psyllids of both haplotypes exhibit lower fitness on bittersweet nightshade than on potato, which may limit dispersal between these hosts (Mustafa et al., 2015). Overall, because western psyllids rarely colonized nightshade, and northwestern psyllids were largely absent from potato, management of potato psyllids in the northwestern US should focus on mapping and controlling patches of matrimony vine rather than considering bittersweet nightshade.

Despite finding that the W and NW ADMIXTURE genetic groups are distinct populations with limited gene flow, we identified several hybrids from inconsistencies between genetic groups identified using COI versus SNP‐based markers (e.g., Wosula, Chen, Fei, & Legg, 2017). While the mitochondrial COI marker is commonly used to delimit psyllid haplotypes (e.g., Swisher, Munyaneza, & Crosslin, 2012, 2013a; Swisher et al., 2013b, 2014), it is a single, maternally inherited locus and therefore often provides insufficient genetic resolution to distinguish populations and, furthermore, cannot reveal gene flow between populations (e.g.,Ballard & Whitlock, 2004; Collins & Cruickshank, 2013; Dupuis, Roe, & Sperling, 2012; Hurst & Jiggins, 2005; Pinto et al., 2014; Wosula et al., 2017). Our study highlights the need to use caution when relying solely on COI‐defined genetic groups and strengthens the argument for developing SNP‐based assays to define closely related genetic groups where interbreeding may occur (e.g., Chapman et al., 2015).

Furthermore, we found several lines of evidence suggesting that there may be an ongoing cytoplasmic incompatibility‐induced sweep of *Wolbachia*, and associated western mitochondrial haplotypes and endosymbionts, into the resident northwestern psyllid genetic group. First, there appears to be high prevalence of *Wolbachia* in the field, as over 90% of the W ADMIXTURE genetic group psyllids harbored *Wolbachia*. Second, over 90% of hybrid psyllids were of the western COI haplotype but more genetically similar to the northwestern haplotype, indicating that most interbreeding events were between *Wolbachia*‐infected western females and uninfected northwestern males. This could occur if *Wolbachia* induces cytoplasmic incompatibility, leading to embryonic death in matings between uninfected females and infected males (Werren et al., 2008). In the laboratory, 73% of eggs from crosses between *Wolbachia*‐infected western females and uninfected northwestern males are viable, whereas only 2% of eggs from crosses between uninfected northwestern females and infected western males are viable (Cooper et al., 2015). Interestingly, there were two hybrids that appear to be from the reciprocal mating (northwestern female and western male) (Table S2). These hybrids were likely from matings between uninfected northwestern females and uninfected western males that result from imperfect vertical transmission of *Wolbachia* (Figure 6). Altogether, the high prevalence of *Wolbachia* in western COI‐haplotype psyllids, and the reproductive advantage of infected over uninfected females (Cooper et al., 2015), suggest that *Wolbachia* may drive the western matriline through the resident northwestern genetic group of psyllids. However, the small number of hybrids sampled, and single year of sampling, make it impossible to conclusively determine whether such a sweep is occurring, or if it is even possible under these particular conditions. Continued in‐depth sampling throughout the region, as well as complementary modeling approaches (e.g., Telschow, Hammerstein, & Werren, 2002; Hancock et al., 2011), are necessary to determine whether such a sweep may be taking place in the northwestern US.

Our study did not examine transmission of the zebra chip pathogen by the two genetic groups or their hybrids. However, this could be a fruitful area for future inquiry as hybridization has the potential to alter disease dynamics. Psyllids of the western COI haplotype are the predominant vector of the zebra chip pathogen in the region, while the LSO bacterium is less frequently found in northwestern COI haplotypes (Swisher et al., 2013a, 2014). Endosymbionts may play a role in this haplotype‐specific pathogen transmission, as has been shown for other insect‐vectored pathosystems (Chuche, Auricau‐Bouvery, Danet, & Thiéry, 2017; Su et al., 2013; Weiss & Aksoy, 2011). We found that the endosymbionts of hybrid psyllids were generally characteristic of the western COI haplotype, although hybrid psyllids were more similar to the NW ADMIXTURE genetic group (Figure 6). If the efficacy of western psyllids as vectors of the zebra chip pathogen is tied closely with maternally inherited genes or their unique endosymbionts, then the *Wolbachia*‐mediated introgression of genes from western into resident northwestern psyllid populations could ultimately lead to greater incidence of zebra chip disease in potato. However, before such conclusions can be made, future work is needed to evaluate whether (a) such a genetic sweep is indeed occurring (as noted above), (b) western psyllids are superior vectors of the zebra chip pathogen, (c) this enhanced transmission is linked to particular maternally inherited genes or particular endosymbionts, and (d) if hybrids also exhibit enhanced transmission. More generally, we recommend that future work should examine any impacts that endosymbionts other than *Wolbachia* have on potato psyllids’ ability to feed on different host plants, and if this in turn impacts pathogen transmission. Indeed, there is preliminary evidence from several other psyllid species that these endosymbionts might play an important role (Subandiyah et al., 2000; Fromont, Riegler, & Cook, 2016), and endosymbionts’ importance for host physiology, ecology, and evolution are well known for other herbivorous insects (Oliver, Degnan, Burke, & Moran, 2010; Wilson & Duncan, 2015).

Overall, our results suggest that both host–plant associations and *Wolbachia* are shaping the genetic integration of putatively introduced and resident populations of a plant pathogen vector, the potato psyllid. In doing so, these factors might have the potential to alter regional vector capacity and zebra chip outbreaks. While future work is necessary to elucidate mechanisms and to confirm that such a trend is indeed taking place in this system, our work highlights the potential importance of incorporating vector genetic structure, host–plant associations, and endosymbionts to understand and predict disease dynamics in other systems. Understanding interactions among these factors and their impact on disease dynamics is increasingly important, as changing climate and land‐use practices will continue to bring allopatric insect vector populations together, with potentially dire consequences for disease outbreaks.

## Supporting information

Supplementary Material

## Data Availability

The raw reads of NextRAD and microbiome were deposited in the NCBI sequence read archive with BioProject ID PRJNA401036. Scripts used to analyze microbiome data are available in the leading author's GitHub repo https://github.com/zhen-fu/psyllid_16S.
