# Supplementary material for: Host plants and Wolbachia shape the population genetics of sympatric herbivore populations
Source: Evol Appl. 2020 Sep 12;13(10):2740–53. doi: 10.1111/eva.13079 (PMC7691456; doi:10.1111/eva.13079)

Supporting Information for:

Host plants and endosymbionts shape the population genetics of sympatric herbivore populations

Table of Contents:

**Table S1**	Page 2-3
**Table S2**	Page 4-7
**Table S3**	Page 8
**Table S4**	Page 9
**Table S5**	Page 10
**Table S6**	Page 11-12
**Fig S1**	Page 13

Table S1. Detailed sampling information

Site name	Coordinates	Plant host	# of sampling dates	# of psyllids processed
Day	44° 29' 22" N, 119° 35' 1" W	Matrimony vine	2	20
Mit	44° 34' 26" N, 120° 10' 19" W	Matrimony vine	2	19
Har	43° 38' 41" N, 116° 12' 31" W	Matrimony vine	2	19
Eag	43° 43' 23" N, 116° 19' 10" W	Matrimony vine	2	19
Gra	42° 34' 17" N, 114° 30' 0" W	Matrimony vine	1	5
Vale	43° 59' 13" N, 117° 14' 41" W	Matrimony vine	1	6
ProL	46° 13' 25" N, 119° 47' 24" W	Matrimony vine	2	19
Kah	46° 39' 22" N, 118° 29' 46" W	Matrimony vine	2	18
Pasco	46° 30' 6" N, 118° 47' 32" W	Matrimony vine	2	18
Selah	46° 40' 45" N ,120° 31' 51"W	Matrimony vine	2	17
PuL	46° 43' 33" N, 117° 10' 3" W	Matrimony vine	2	19
Lichty	46° 17' 40" N, 119° 57' 6" W	Nightshade	2	19
ProN	46° 14' 16" N, 119° 49' 14" W	Nightshade	2	17
Buena	46° 25' 21" N, 120° 18' 24" W	Nightshade	2	15
CX	46° 50' 51" N, 117° 28' 44" W	Nightshade	2	20
Mesa	46° 35' 18" N, 119° 0' 1" W	Nightshade	2	19
FH	46° 59' 55" N, 119° 41' 5" W	Nightshade	2	19
PuN	46° 43' 40" N, 117° 10' 24" W	Nightshade	2	16
BSND	43° 40' 10" N, 116° 28' 21" W	Nightshade	2	15
Die	42° 35' 47" N, 114° 23' 20" W	Nightshade	1	10
LB1	45° 52' 51" N, 119° 3' 13" W	Nightshade	1	9
MES_N	43° 58' 51" N, 117° 1' 19" W	Nightshade	1	10
Picabo	43° 18' 54" N, 114° 7' 51" W	Nightshade	1	8
L11	42° 57' 3" N, 115° 32' 41" W	Potato	1	4
L5	43° 35' 30" N, 116° 44' 35" W	Potato	1	10
L1	43° 42' 43" N, 117° 0' 8" W	Potato	1	10
L26	42° 30' 6" N, 114° 26' 9" W	Potato	1	9
L8	43° 24' 40" N, 116° 38' 14" W	Potato	1	10
L24	42° 35' 10" N, 114° 18' 4" W	Potato	1	9
L32	42° 30' 34" N, 114° 37' 43" W	Potato	1	10
Tr10	45° 44' 53" N, 119° 9' 19" W	Potato	1	10
Tr5	45° 52' 31" N, 119° 7' 1" W	Potato	1	8
Tr25	45° 47' 5" N, 119° 48' 21" W	Potato	1	8
Tr16	45° 54' 3" N, 119° 25' 44" W	Potato	1	10
W8	46° 41' 11" N, 119° 52' 26" W	Potato	1	10
W10	47° 11' 34" N, 119° 51' 11" W	Potato	1	10
N8	46° 51' 14" N, 119° 4' 49" W	Potato	1	10

N2	47° 8' 41" N, 119° 3' 57" W	Potato	1	6
N7	46° 43' 45" N, 119° 0' 15" W	Potato	1	10
NC5	46° 55' 30" N, 119° 38' 34" W	Potato	1	9
S2	45° 56' 45" N, 119° 51' 2" W	Potato	1	9
S9	46° 1' 35" N, 119° 18' 6" W	Potato	1	10
SE2	46° 12' 36" N, 118° 41' 36" W	Potato	1	10
C4	46° 26' 19" N, 119° 2' 23" W	Potato	1	10
C9	46° 28' 36" N, 119° 13' 20" W	Potato	1	9
MES_P	43° 58' 51" N, 117° 1' 19" W	Potato	1	9

Table S2 ADMIXTURE membership values (K=2), COI-haplotype, and genetic groups of the psyllid samples presented in fineRADstructure plot (N=124[†])

Sample ID	COI-haplotype	ADMIXTURE K=2[‡]	Genetic group	Position in FineRADstructure
LB12	western	0.6778	W genetic group	lower square
W82	western	0.7238	W genetic group	lower square
NC55	western	0.6946	W genetic group	lower square
L24_6	western	0.6996	W genetic group	lower square
CX_Sep_5	western	0.7571	W genetic group	lower square
SE28	western	0.723	W genetic group	lower square
Selah_Sep8	western	0.777	W genetic group	lower square
S92	western	0.7616	W genetic group	lower square
LB11	western	0.6976	W genetic group	lower square
LB15	western	0.7058	W genetic group	lower square
S22	western	0.7423	W genetic group	lower square
C44	western	0.7332	W genetic group	lower square
ProL_May5	western	0.7299	W genetic group	lower square
Day_Jul_2	western	0.7465	W genetic group	lower square
ProL_Sep1	western	0.7441	W genetic group	lower square
L26_3	western	0.7242	W genetic group	lower square
C48	western	0.7364	W genetic group	lower square
Har_Jun_4	western	0.5239	W genetic group	lower square
Mit_Sep_2	western	0.5787	W genetic group	lower square
Mit_Jul_9	northwestern	0.5109	W genetic group	lower square
ProL_May1	northwestern	0.5255	W genetic group	lower square
Kah_May2	western	0.529	W genetic group	lower square
Kah_May5	western	0.5468	W genetic group	lower square
Pasco_May9	western	0.5499	W genetic group	lower square
Pasco_May3	western	0.5044	W genetic group	lower square
Day_Jul_1	western	0.4998	NW genetic group	lower square
Day_Jul_3	western	0.5621	W genetic group	lower square
Day_Jul_7	western	0.5184	W genetic group	lower square
Mit_Jul_10	western	0.5382	W genetic group	lower square
Mit_Jul_1	western	0.5422	W genetic group	lower square
NC51	western	0.5643	W genetic group	lower square
Day_Jul_6	western	0.6331	W genetic group	lower square
LB17	western	0.6325	W genetic group	lower square
L8_3	western	0.6267	W genetic group	lower square
W87	western	0.6174	W genetic group	lower square
NC54	western	0.653	W genetic group	lower square

Tr169	western	0.5858	W genetic group	lower square
Tr167	western	0.6802	W genetic group	lower square
Mit_Jul_3	western	0.6675	W genetic group	lower square
Pasco_May8	western	0.617	W genetic group	lower square
Selah_May3	western	0.7013	W genetic group	lower square
Selah_Sep4	western	0.6457	W genetic group	lower square
Mit_Sep_6	western	0.6025	W genetic group	lower square
ProL_May7	western	0.6149	W genetic group	lower square
Pasco_May5	western	0.5935	W genetic group	lower square
Tr166	western	0.6019	W genetic group	lower square
L5_3	western	0.8324	W genetic group	lower square
LB18	western	0.8096	W genetic group	lower square
N74	western	0.8192	W genetic group	lower square
L1_7	western	0.9903	W genetic group	lower square
MES_P9	western	0.9166	W genetic group	lower square
N89	western	0.9616	W genetic group	lower square
SE26	western	0.885	W genetic group	lower square
Tr162	western	0.9555	W genetic group	lower square
Pasco_May2	western	0.9276	W genetic group	lower square
SE210	western	0.9729	W genetic group	lower square
Kah_Sep4	western	0.9287	W genetic group	lower square
W86	western	0.9715	W genetic group	lower square
S93	western	0.9637	W genetic group	lower square
Kah_Sep2	western	0.9034	W genetic group	lower square
S99	western	0.9986	W genetic group	lower square
L24_3	western	0.8447	W genetic group	lower square
C47	western	0.9501	W genetic group	lower square
Har_Sep_9	western	0.9125	W genetic group	lower square
ProL_May3	western	0.9126	W genetic group	lower square
Pasco_Sep3	western	0.92	W genetic group	lower square
Tr58	western	0.8261	W genetic group	lower square
Day_Sep_2	western	0.8583	W genetic group	lower square
S97	western	0.9002	W genetic group	lower square
Day_Sep_8	western	0.9614	W genetic group	lower square
L8_8	western	1	W genetic group	lower square
Kah_Sep1	western	1	W genetic group	lower square
L26_5	western	1	W genetic group	lower square
W109	western	1	W genetic group	lower square
Tr101	western	1	W genetic group	lower square
CX_Sep_1	western	0.2449	NW genetic group	upper square
CX_Sep_6	western	0.3027	NW genetic group	upper square

CX_Sep_3	western	0.2303	NW genetic group	upper square
Eag_Jun_6	western	0.2082	NW genetic group	upper square
Har_Sep_5	northwestern	0.2574	NW genetic group	upper square
Selah_Sep1	northwestern	0.2219	NW genetic group	upper square
Har_Jun_3	northwestern	0.2184	NW genetic group	upper square
Har_Jun_9	northwestern	0.2121	NW genetic group	upper square
Har_Jun_10	northwestern	0.1858	NW genetic group	upper square
Buena_Jun_3	northwestern	0.1836	NW genetic group	upper square
Eag_Sep_5	northwestern	0.1991	NW genetic group	upper square
ProN_Sep_5	northwestern	0.2317	NW genetic group	upper square
Selah_May1	northwestern	0.1403	NW genetic group	upper square
Selah_Sep7	northwestern	0.2113	NW genetic group	upper square
Mit_Jul_5	western	0.4595	NW genetic group	upper square
W101	western	0.4201	NW genetic group	upper square
ProL_May9	western	0.4194	NW genetic group	upper square
Pasco_May1	western	0.4908	NW genetic group	upper square
Kah_May6	western	0.4149	NW genetic group	upper square
Pasco_May4	northwestern	0.465	NW genetic group	upper square
CX_Sep_2	western	0.5089	W genetic group	upper square
CX_Sep_10	northwestern	0.4613	NW genetic group	upper square
Kah_May10	western	0.3689	NW genetic group	upper square
Tr164	western	0.3136	NW genetic group	upper square
Har_Jun_5	northwestern	0.2976	NW genetic group	upper square
Selah_May7	western	0.3477	NW genetic group	upper square
Buena_Sep_3	northwestern	0.0966	NW genetic group	upper square
Lichty_Sep_5	western	0.0929	NW genetic group	upper square
ProN_Sep_3	western	0.1135	NW genetic group	upper square
BSND_Jun_3	northwestern	0	NW genetic group	upper square
Picabo3	western	0.0876	NW genetic group	upper square
Har_Jun_2	western	0.0756	NW genetic group	upper square
Picabo8	northwestern	0.0104	NW genetic group	upper square
Eag_Jun_8	northwestern	0.0378	NW genetic group	upper square
W105	western	0.1038	NW genetic group	upper square
Selah_May5	northwestern	0.0455	NW genetic group	upper square
Buena_Sep_1	northwestern	0.0375	NW genetic group	upper square
Lichty_Jun_1	northwestern	0.0462	NW genetic group	upper square
Buena_Sep_10	northwestern	0.0014	NW genetic group	upper square
ProN_Jun_1	northwestern	0	NW genetic group	upper square
Lichty_Sep_6	western	0	NW genetic group	upper square
FH_Jul_4	northwestern	0	NW genetic group	upper square
ProN_Jun_9	northwestern	0.0295	NW genetic group	upper square

FH_Sep_5	northwestern	0	NW genetic group	upper square
Selah_Sep6	northwestern	0.0553	NW genetic group	upper square
Die_Jun_4	northwestern	0	NW genetic group	upper square
Mit_Jul_6	northwestern	0.015	NW genetic group	upper square
PuN_Sep_6	northwestern	0	NW genetic group	upper square
PuL_May7	northwestern	0	NW genetic group	upper square

†The order of the samples was in corresponding order of samples from left to right in Fig. 4B.
‡samples with ADMIXTRUE K > 0.5 was designated as W genetic group, otherwise NW genetic group.

Table S3. Results of multiple matrix regression with randomization (MMRR)

	Individual explainable variable					
	Host plant		COI-haplotype		Geographic distance	
**r-squared**	0.226		0.652		0.018	
**coefficient**	Intercept	Host plant	Intercept	haplotype	Intercept	Geo distance
	0.000	0.570	0.000	0.808	0.000	0.135
**t-statistics**	Intercept	Host plant	Intercept	haplotype	Intercept	Geo distance
	0.000	19.820	0.000	39.182	0.000	3.887
**t-pvalue**	Intercept	Host plant	Intercept	haplotype	Intercept	Geo distance
	0.001	0.001	0.001	0.001	0.429	0.052
**F-statistic**	238.7618		1535.252		15.107	
**p-value**	0.001		0.001		0.052	

Methods for MMRR:
Following the instructions in Wang (2013), we picked populations that had sample size ≥ 8, consistent with our F_{ST} estimation (see main manuscript). Given that we were only considering the explainable variables of geographic distance, host plants, and haplotypes, we merged psyllids that were collected at two time points from the same location into one population. For host plants, we created a matrix with the following distance designation: a pair of populations from the same host=0, a pair of populations from different hosts = 1. For haplotype matrix, if a population had comparable numbers of both W and NW haplotyped individuals, that population was excluded. This criterion excluded four populations. To create a haplotype matrix, we used the following designation, a pair of populations of the same haplotype = 0, a pair of populations with different haplotypes = 1 (only two haplotypes, NW and W were used here). Geographic distance was calculated using R package geodist. F_{ST} was calculated with R package StAMPP.

Table S4. F_{ST} of non-crop host sites with two sampling points

Sampling site	F_{ST}	Host	Region	% of NW genetic group in pre-season	% of NW genetic group in post-season
BSND	-0.003	Nightshade	Snake River Plain	100.0%	90.0%
Buena	-0.013	Nightshade	Columbia Basin	100.0%	100.0%
Day	0.034*	Matrimony vine	Non-growing region	0.0%	0.0%
Eag	0.123*	Matrimony vine	Snake River Plain	100.0%	25.0%
FH	-0.008	Nightshade	Columbia Basin	100.0%	100.0%
Har	0.089*	Matrimony vine	Snake River Plain	90.0%	28.6%
Kah	0.039*	Matrimony vine	Columbia Basin	25.0%	0.0%
Lichty	0.05*	Nightshade	Columbia Basin	100.0%	100.0%
Mesa	-0.004	Nightshade	Columbia Basin	100.0%	90.0%
Mit	0.056*	Matrimony vine	Non-growing region	30.0%	0.0%
Pasco	0.027*	Matrimony vine	Columbia Basin	12.5%	0.0%
ProL	0.013*	Matrimony vine	Columbia Basin	11.1%	0.0%
ProN	0.012*	Nightshade	Columbia Basin	100.0%	100.0%
PuL	0.015*	Matrimony vine	Non-growing region	100.0%	77.8%
PuN	0.039*	Nightshade	Non-growing region	100.0%	100.0%
Selah	0.047*	Matrimony vine	Columbia Basin	87.5%	37.5%

*indicates the F_{ST} value was significant ($p < 0.05$) between pre- and post-season populations of that sampling site from 500 bootstraps.

Table S5. BLAST results of seven unique strains of microbiome associated with *Bactericera cockerelli* samples

Seq ID	Family	Genus	species	Top match to genes in GenBank (GenBank ID)	Identity
W. pipientis-1	Anaplasmataceae	*Wolbachia*	*pipentis*	*Wolbachia pipientis* 16S ribosomal RNA gene (AF501664.1)	100%
W. pipientis-2[*]	Anaplasmataceae	*Wolbachia*	*pipentis*	*Wolbachia* sp. isolate 37_NW2_EstivaGerbiSP 16S ribosomal RNA gene (MG977008.1)	100%
C. ruddii-1	NA	*Candidatus* Carsonella	*ruddii*	*Candidatus* Carsonella ruddii clone 2 16S ribosomal RNA gene, partial sequence (KR045612.1)	100%
C. ruddii-2[#]	NA	*Candidatus* Carsonella	*ruddii*	Carsonella ruddii natural-host *Bactericera cockerelli* tryptophanyl-tRNA synthetase (AF211126.2)	99.75%
Entero-1	Enterobacteriaceae	unknown	unknown	Secondary endosymbiont of *Bactericera cockerelli* 16S ribosomal RNA gene ([1]AF263557.1)	100%
Entero-2	Enterobacteriaceae	unknown	unknown	Secondary endosymbiont of *Bactericera cockerelli* 16S ribosomal RNA gene ([1]AF263557.1)	99.5%
Entero-3	Enterobacteriaceae	unknown	unknown	Secondary endosymbiont of *Ctenarytaina eucalypti*, complete genome ([2]CP003546.1)	94.83%

[*] *W. pipientis*-2 is one nucleotide different from *W. pipientis*-1
[#] C. ruddii-2 is three nucleotides different form C. ruddii-1
[1] AF263557.1 was derived from a secondary endosymbiont of *Bactericera cockerelli* without further taxonomic information available (Thao et al. 2000).
[2] CP003546.1 was a sequence derived from a secondary endosymbiont of *Ctenarytaina eucalypti* (blue gum psyllids) that was not further classified (Sloan & Moran, 2008)

Sloan DB, Moran NA. 2012. Genome reduction and co-evolution between the primary and secondary bacterial symbionts of psyllids. *Molecular Biology and Evolution* 29(12):3781–3792. doi:10.1093/molbev/mss180

Thao ML, Clark MA, Baumann L, Brennan EB, Moran NA, Baumann P. 2000. Secondary endosymbionts of psyllids have been acquired multiple times. *Current Microbiology* 41(4):300–304. doi:10.1007/s002840010138

Table S6. Relative abundance of bacteria that were associated with 77 potato psyllid samples

Sample.ID	Host	COI.hap	Genetic group	tax1[#]	tax2	tax3	tax4	tax5	tax6	tax7	other taxa
Selah_May1	M	NW	NW ADM	0	0	0.056	0	0	0.863	0.036	0.045
Selah_May2	M	NW	NW ADM	0	0	0.159	0	0	0.706	0.120	0.015
Selah_May4	M	NW	NW ADM	0	0	0.123	0	0	0.656	0.220	0.001
Selah_May6	M	NW	NW ADM	0	0	0.111	0	0	0.784	0.104	0.001
Selah_May9	M	NW	NW ADM	0	0	0.201	0	0	0.764	0.033	0.002
Selah_Sep1	M	NW	NW ADM	0	0	0.176	0	0	0.624	0.198	0.002
Selah_Sep6	M	NW	NW ADM	0	0	0.471	0	0	0.410	0.110	0.009
PuL_May2	M	NW	NW ADM	0	0	0.159	0	0	0.745	0.088	0.008
PuL_May3	M	NW	NW ADM	0	0	0.106	0	0	0.780	0.107	0.007
PuL_May4	M	NW	NW ADM	0	0	0.182	0	0	0.653	0.152	0.014
PuL_May5	M	NW	NW ADM	0	0	0.193	0	0	0.729	0.072	0.006
PuL_May6	M	NW	NW ADM	0	0	0.115	0	0	0.824	0.045	0.015
PuL_May7	M	NW	NW ADM	0	0	0.132	0	0	0.790	0.073	0.004
PuL_May8	M	NW	NW ADM	0	0	0.196	0	0	0.693	0.111	0.001
Pasco_Ma4	M	NW	NW ADM	0	0	0.191	0	0	0.684	0.123	0.001
PuL_Aug1	M	NW	NW ADM	0	0	0.094	0	0	0.760	0.001	0.144
PuL_Aug4	M	NW	NW ADM	0	0	0.085	0	0	0.569	0.341	0.005
PuN_Jul5	N	NW	NW ADM	0	0	0.139	0	0	0.834	0.025	0.001
PuN_Sep6	N	NW	NW ADM	0	0	0.144	0	0	0.824	0.026	0.006
PuN_Sep8	N	NW	NW ADM	0	0	0.100	0	0	0.801	0.089	0.011
PuN_Sep9	N	NW	NW ADM	0	0	0.215	0	0	0.741	0.036	0.008
Eag_Jun_1	M	NW	NW ADM	0	0	0.168	0	0	0.734	0.096	0.002
Eag_Jun_8	M	NW	NW ADM	0	0	0.134	0	0	0.856	0	0.009
Pasco_Sep3	M	W	W ADM	0.616	0.009	0	0.018	0.308	0	0.022	0.027
Pasco_Sep4	M	W	W ADM	0.602	0.009	0	0.024	0.336	0	0.014	0.014
Pasco_Sep5	M	W	W ADM	0.901	0	0	0.011	0.070	0	0.008	0.010
Pasco_Sep7	M	W	W ADM	0.782	0.012	0	0.012	0.153	0	0.017	0.025
Pasco_Sep8	M	W	W ADM	0.902	0	0	0.028	0.037	0	0.011	0.023
Pasco_Sep9	M	W	W ADM	0.609	0	0	0.025	0.355	0	0.002	0.009
Selah_May3	M	W	W ADM	0.915	0.013	0	0.018	0.013	0	0.025	0.016
Pasco_Ma2	M	W	W ADM	0.807	0.014	0	0.064	0.099	0	0.004	0.012
Selah_Sep3	M	W	W ADM	0.693	0.010	0	0.027	0.258	0	0.002	0.009
Selah_Sep5	M	W	W ADM	0.411	0	0	0.048	0.495	0	0.034	0.012
Mit_Jul_2	M	W	W ADM	0.722	0	0	0.042	0.213	0	0.003	0.019
W105	P	W	NW ADM	0	0	0.080	0	0	0.852	0.058	0.009
Mit_Jul_5	M	W	W ADM	0.959	0.016	0	0.004	0.008	0	0.001	0.012
Mit_Sep_1	M	W	W ADM	0.913	0.014	0	0.006	0.051	0	0.011	0.006
Pasco_Ma3	M	W	W ADM	0.877	0.015	0	0.090	0.001	0	0.001	0.015
Mit_Sep2	M	W	W ADM	0.716	0.011	0	0.020	0.216	0	0.030	0.008
Mit_Sep4	M	W	W ADM	0.877	0.014	0	0.011	0.086	0	0.005	0.007

Mit_Sep6	M	W	W ADM	0.704	0.010	0	0.012	0.225	0	0.041	0.009
PuL_Aug3	M	W	W ADM	0.899	0	0	0.004	0.082	0	0.008	0.007
C92	P	W	W ADM	0.973	0	0	0.023	0	0	0	0.004
C95	P	W	W ADM	0.882	0	0	0.006	0.100	0	0.005	0.008
Eag_Jun6	M	W	NW ADM	0.964	0	0	0.016	0.019	0	0	0.001
LB16	N	W	W ADM	0.697	0	0	0.080	0.203	0	0.014	0.005
MES_N3	N	W	W ADM	0.821	0	0	0.031	0.124	0	0.016	0.008
MES_N4	N	W	W ADM	0.767	0	0	0.058	0.138	0	0.028	0.009
MES_N7	N	W	W ADM	0.854	0.014	0	0.009	0.116	0	0	0.007
L24_6	P	W	W ADM	0.893	0	0	0.012	0.087	0	0.008	0.001
Tr165	P	W	W ADM	0.757	0.012	0	0.030	0.190	0	0.003	0.008
Pasco_Ma7	M	W	W ADM	0.798	0.013	0	0.036	0.119	0	0.015	0.019
Tr166	P	W	W ADM	0.001	0	0	0.200	0.758	0	0.035	0.006
Tr167	P	W	W ADM	0.824	0	0	0.012	0.152	0	0.004	0.009
Tr169	P	W	W ADM	0.001	0	0	0.159	0.806	0	0.033	0.002
W109	P	W	W ADM	0.777	0	0	0.020	0.186	0	0.008	0.010
C91	P	W	W ADM	0.757	0	0	0.024	0.203	0	0.007	0.009
MES_P5	P	W	W ADM	0.880	0	0	0.011	0.102	0	0.002	0.006
L5_2	P	W	W ADM	0.886	0	0	0.028	0.073	0	0.010	0.002
L5_3	P	W	W ADM	0.866	0	0	0.012	0.110	0	0.005	0.007
L5_4	P	W	W ADM	0.891	0	0	0.007	0.094	0	0.006	0.002
Lichty_Jun6	N	W	NW ADM	0.831	0	0	0.005	0.126	0	0	0.039
L5_5	P	W	W ADM	0.794	0	0	0.014	0.185	0	0	0.007
L5_6	P	W	W ADM	0.781	0	0	0.018	0.191	0	0.002	0.008
L24_3	P	W	W ADM	0.954	0	0	0.014	0.027	0	0.003	0.002
L24_4	P	W	W ADM	0.829	0	0	0.032	0.132	0	0.002	0.006
L24_5	P	W	W ADM	0.764	0	0	0.008	0.223	0	0	0.005
L24_7	P	W	W ADM	0.878	0	0	0.005	0.108	0	0.005	0.004
L24_8	P	W	W ADM	0.823	0	0	0.005	0.156	0	0.010	0.006
L249	P	W	W ADM	0.827	0	0	0.014	0.141	0	0.012	0.006
Pasco_Ma9	M	W	W ADM	0.002	0	0	0.498	0.334	0	0.153	0.013
Pasco_Sep1	M	W	W ADM	0.663	0.011	0	0.021	0.280	0	0.008	0.018
Pasco_Ma6	M	W	NA*	0.764	0.012	0	0.020	0.177	0	0.013	0.014
L5_8	P	W	NA*	0.941	0	0	0.023	0.020	0	0.012	0.003
L5_9	P	W	NA*	0.610	0	0	0.023	0.109	0	0.012	0.246
MES_P8	P	W	NA*	0.808	0	0	0.030	0.145	0	0.017	0.001
Pasco_Sep2	M	W	NA*	0.732	0.011	0	0.014	0.214	0	0.013	0.016

*Indicates samples without genotyping information due to low quality reads

taxon 1 to 7 were *W. pipientis*-1, *W. pipientis*-2, C. ruddii-1, C. ruddii-2, Entero-1, Entero-2, and Entero-3 respectively; other taxa were low abundance taxa that fell out the filtering criteria (see main text).

Fig S1. f_3 statistic indicating individual hybrids (y; W ADMIXTURE genetic group, NW ADMIXTURE genetic group). Negative f_3 indicates that individual y is admixed between two source populations (putative introduced and resident populations). Colors represent host plants from which potato psyllid samples were collected. Error bars represent standard errors. *indicates six individuals were also flagged as possible hybrids from at least one of the three tests: fineRADstructure, ADMIXTURE, and PCA.

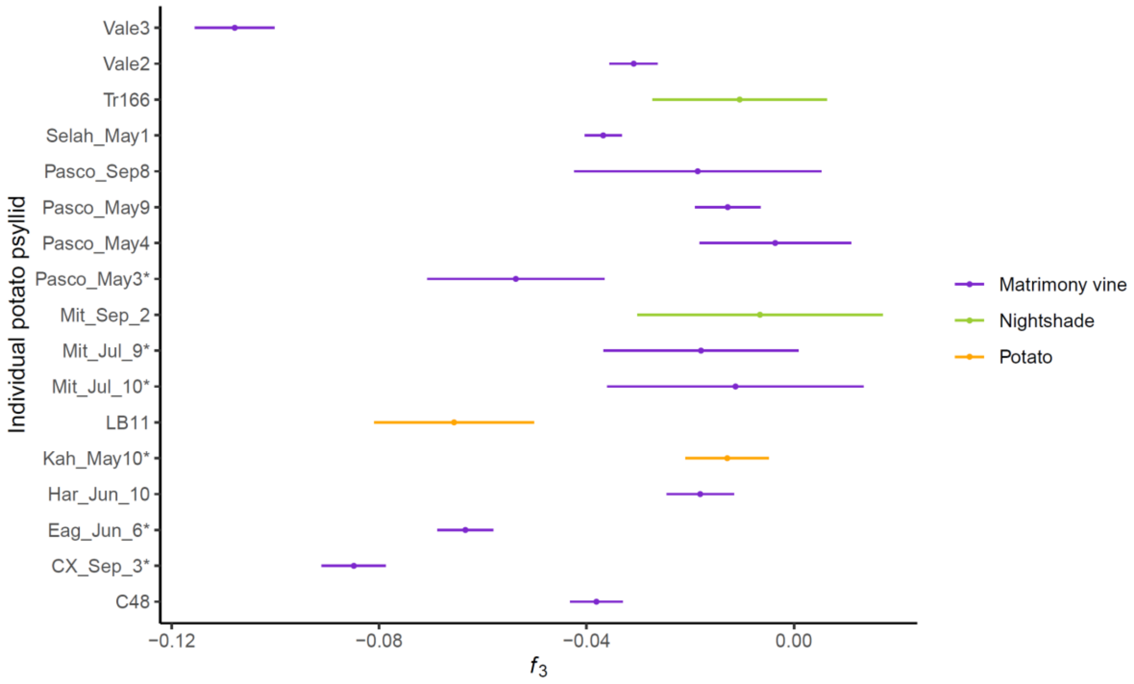

Supplement: Supplementary file 1 — Supplementary Material [file EVA-13-2740-s001.pdf]
